# The Impact of Behavioral Intervention on Obesity Mediated Declines in Mobility Function: Implications for Longevity

**DOI:** 10.4061/2011/392510

**Published:** 2011-10-16

**Authors:** Joe Nocera, Thomas W. Buford, Todd M. Manini, Kelly Naugle, Christiaan Leeuwenburgh, Marco Pahor, Michael G. Perri, Stephen D. Anton

**Affiliations:** ^1^Department of Veterans Affairs, Rehabilitation Research and Development, Brain Rehabilitation Research Center Malcom Randall VA Medical Center, Gainesville, FL 32608, USA; ^2^Department of Aging and Geriatric Research, University of Florida, Gainesville, FL 32611, USA; ^3^Department of Clinical and Health Psychology, University of Florida, Gainesville, FL 32610, USA

## Abstract

A primary focus of longevity research is to identify prognostic risk factors that can be mediated by early treatment efforts. To date, much of this work has focused on understanding the biological processes that may contribute to aging process and age-related disease conditions. Although such processes are undoubtedly important, no current biological intervention aimed at increasing health and lifespan exists. Interestingly, a close relationship between mobility performance and the aging process has been documented in older adults. For example, recent studies have identified functional status, as assessed by walking speed, as a strong predictor of major health outcomes, including mortality, in older adults. This paper aims to describe the relationship between the comorbidities related to decreased health and lifespan and mobility function in obese, older adults. Concurrently, lifestyle interventions, including diet and exercise, are described as a means to improve mobility function and thereby limit the functional limitations associated with increased mortality.

## 1. Introduction

The term longevity can be used to refer to a “long life” for an individual or more broadly to life expectancy within a population. In recent years, scientists have devoted much attention to finding ways to increase longevity. To date, much of this work has focused on understanding the biological processes that may contribute to aging and age-related disease conditions. A number of potential biological targets have been identified during the past few decades, and a wide range of intervention approaches are currently being developed. The types of interventions considered to have the potential to affect the aging process include biochemical and genetic techniques, hormonal treatments, and behavioral approaches to reduce age-related comorbidities and thereby increase mean lifespan. Although biological approaches may have significant potential in the future, the effects of behavioral interventions on age-related conditions can be more immediately evaluated and put into practice at the present time. 

To determine the effectiveness of behavioral interventions and other treatment approaches to increase longevity, a benchmark is needed that enables scientists to determine whether or not the intervention was successful. Because it is unrealistic to conduct lifespan studies in humans, a surrogate endpoint is needed to estimate the effect interventions could have on longevity. Recently, gait (i.e., walking) speed—a simple, valid, and reliable clinical test—was considered to be such an endpoint [1]. A growing body of literature has identified walking speed as a strong predictor of major health outcomes and mortality in older adults [2–4]. In a pooled analysis of nine cohort studies with follow-up periods ranging from 6 to 21 years, declines in gait speed were found to be directly associated with decreased survival [1]. Specifically, hazard ratios for survival revealed that there was a 12% risk reduction in mortality for every 0.10 meters per second increase in gait speed. As recently noted by Cesari [5], the findings of Studenski et al.'s pooled analysis provide the statistical foundations to estimate expected survival in older adults based solely on gait speed [1]. Based on these consistent and robust findings, we argue that gait speed may represent a useful marker of overall health status and may also be a useful prognostic indicator of mean lifespan in older adults. Further, because walking places demands on multiple organ systems and demands on input from the central nervous system, it may serve as the ideal target to evaluate the efficacy of interventions aimed at improving health and increasing lifespan. 

Against the backdrop of these findings on the importance of mobility function, a growing body of literature indicates that obese, older adults are at particularly high risk of functional decline, marked by reductions in mobility (i.e., walking speed). This is of significant concern because the number and proportion of obese, older adults has increased dramatically during the past two decades [6]. Recent estimates indicate that an alarming 35% of older adults are obese and another 33% are overweight, which places them at risk for obesity [6]. This paper describes the key behavioral factors contributing to the development of obesity in older adults, the major pathways through which obesity affects mobility function, and the relationship between important comorbidities related to decreased lifespan and mobility function in obese, older adults. Concurrently, lifestyle interventions, including diet and exercise, targeted toward obese older adults are described as a means to target mobility function and limit the functional limitations associated with increased mortality risk.

## 2. Behavioral Mechanisms of Obesity in Older Adults

Both obesity and sedentary lifestyle appear to contribute to the body composition changes (i.e., increased body fat, decreased muscle mass) that promote age-related functional decline [7–9]. As such, obese, older adults may be particularly susceptible to the adverse effects of weight gain because of the loss of muscle mass that occurs with aging (i.e., sarcopenia) [10, 11]. Loss in muscle mass by itself is associated with impairments in mobility in older adults [12]. Moreover, the combination of muscle loss and fat gain may act synergistically to lead to further reductions in mobility in older adults [13–15]. 

Excessive dietary intake, specifically, is a major factor influencing the overall health and body weight of older adults. Epidemiological studies indicate that per capita energy intake has increased by approximately 300 kcal per day from 1985 to 2000 [16]. Unfortunately, the trend of increasing dietary consumption also coincides with a trend to expend less energy with increasing age [17]. Physical inactivity represents another major contributor to the development of obesity and obesity-related morbidity [18]. Currently, the majority of older adults in the US do not engage in even the minimum physical activity recommendations [19]. Moreover, the Centers for Disease Control and Prevention recently reported that 40% of adults engage in no leisure-time physical activity [19]. This is of particular concern in the elderly as older adults are less active with advancing age [17].

## 3. Impact of Obesity on Declines in Mobility

Obesity poses several threats to mobility during aging. Most notable is the direct effect of excess body weight on movement. As body mass increases, the energy and strength required to move the body increases correspondingly. Specifically, work from the Baltimore Longitudinal Study of Aging showed that total knee generative mechanical work expenditure is higher in older adults with obesity [20]. Similarly, work by Messier et al. [21] demonstrated that the absolute peak vertical ground-reaction forces increase almost directly in proportion with body weight. These factors likely contribute to the slower preferred walking speed consistently found in obese, older adults compared to nonobese, older adults [20]. 

A growing body of evidence also indicates that relative skeletal muscle mass (i.e., skeletal muscle mass/body mass), compared to absolute body mass, is a strong predictor of impairments in mobility performance, such as walking and stair climbing [22, 23]. For example, Janssen and colleagues [22] found that there is an increased likelihood of functional impairment and disability in older adults if muscle loss progresses to the point where skeletal muscle mass, relative to body weight, reaches 30% below the mean for young adults. Alarmingly, 45% of men and 59% of women in this sample of over 4,500 older adults (age ≥ 60 years) were classified as having sarcopenia, as determined by low relative skeletal muscle mass to body weight. Sarcopenia is strongly associated with the development of functional disability and can lead to the loss of independence for afflicted individuals [22, 24]. Typically, older adults lose an average of 1-2% of their muscle mass yearly after the age of 50 [25, 26]. However, recently the loss in muscle strength has been shown to decline at a faster rate (3–5%) than previously thought [27], and it has been argued that muscle weakness is a stronger predictor of these changes than muscle mass [24, 28]. Additionally, the combination of muscle weakness and obesity increases the risk of mobility impairment greater than obesity or muscle weakness alone [29]. The reductions in both muscle size and the function through maximal strength are important factors in the loss of mobility among older adults [30, 31]. Somewhat paradoxically, obese, older adults typically have more absolute total muscle mass and strength than nonobese peers. Despite this fact, obese individuals also have higher amounts of intermuscular adipose tissue, a potential detriment to the muscle's force-generating capacity [32, 33]. Furthermore, the disparity in total muscle mass is typically not sufficient to account for the disparity in body mass, meaning that obese persons have a lower strength to body mass ratio—an important factor in mobility. This paradoxical state of low (relative) muscle mass and high body weight has come to be known as sarcopenic obesity [34, 35]. These findings are particularly salient because both muscular strength and power, particularly in the weight-bearing lower limb muscle groups [36], have been strongly associated with habitual and maximal gait velocities, as well as other measures of physical function (i.e., chair stand time) in community-dwelling, mobility-limited, older adults [37]. Collectively, these works suggest that a low muscle/body mass ratio directly impairs movement among obese, older adults. 

Musculoskeletal pain, often in the form of osteoarthritis, is also associated with obesity and directly impacts the ability of older persons to remain mobile. Complaints of pain in the leg joints related to osteoarthritis, particularly in the knee, are common in older adults. In fact, self-reported joint pain is often cited as the main factor affecting mobility in older adults [38]. Noteworthy, mobility limitations due to joint pain are compounded by obesity [39]. This is significant considering that most adults with knee osteoarthritis have a body mass in the overweight or obese range (body mass index (BMI) ≥ 25 kg/m^2^) [40]. Additionally, the Women's Health and Aging Study found that obesity was a distinct risk factor for substantially increasing the risk of mobility disability among individuals with chronic pain [41]. In line with this, obesity has been identified as the main preventable risk factor for the onset and progression of osteoarthritis and the associated pain by some experts [42]. Importantly, individuals with chronic pain often modify and lessen their activity by walking and engaging in less physically demanding activities. As such, the age-related degeneration and the associated pain, which is compounded with obesity, can lead to significant reductions in activity. 

In addition to the direct and immediate effects that excess body weight has on movement, obesity may have an overlooked, long-term impact on mobility function through the development of comorbid disease conditions. The development of such conditions, including cardiovascular disease and diabetes, may lead to mobility impairments beyond those observed during obesity alone. In fact, accumulating evidence indicates that older adults with diabetes, obese or not, experience more severe losses of muscle mass and strength than those who are not diabetic [43]. Although the mechanisms that cause this accelerated loss of mobility among older adults who have diabetes are unclear, much of the decline may be due to exacerbated changes in body composition typically observed in advanced age and/or obesity. 

**Figure 1:**
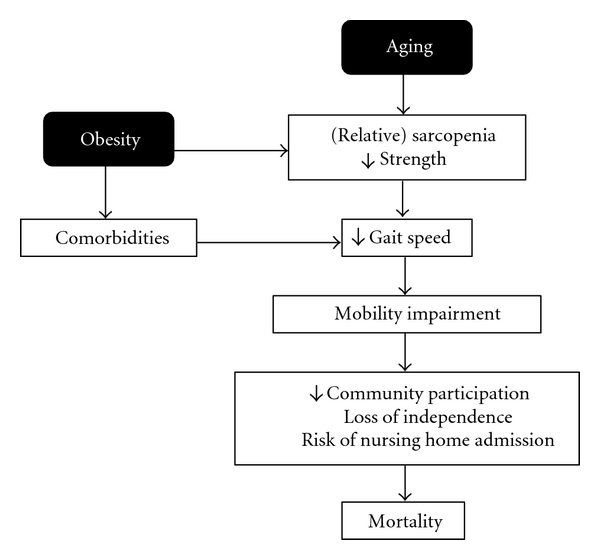
Conceptual model illustrating how obesity potentiates age-related declines in gait speed that might lead to mobility impairment, loss of independence, and mortality.

**Figure 2:**
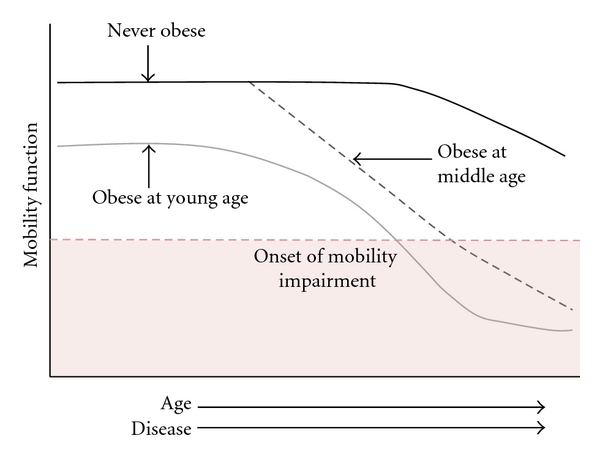
Theoretical illustration demonstrating the relative impact of obesity on mobility function within the context of aging and disease onset.

Collectively, studies to date indicate that excessive body weight, metabolic disease, and sarcopenia all contribute to declines in mobility function (see Figures 1 and 2) [1]. Significantly, comorbidities associated with metabolic disease and sarcopenia are prevalent in older adults, and the health consequences associated with each condition are compounded by obesity (Figure 1). Each of these conditions is directly associated with lifestyle habits. As previously discussed, excessive caloric intake and a sedentary lifestyle promote weight gain and contribute to the progression of age-related sarcopenia and declines in mobility function. Over time, these changes can accelerate the development of mobility impairments and disability (Figure 2). Although aging is the primary risk factor for both of these conditions, healthy dietary and physical activity habits have demonstrated efficacy in attenuating the progression of these conditions. In the next section, we will highlight important research findings over the past few decades demonstrating the beneficial effects of nutritional and exercise interventions on mobility function in obese, older adults.

## 4. Diet and Exercise Interventions to Improve Mobility Function in Obese Older Adults

A current challenge for clinicians and researchers working with overweight, older adults is to design lifestyle-based interventions that can produce significant weight loss while minimizing the loss of fat-free mass. Concerns about weight loss in obese, older adults relate to the documented declines in dynamic force production capability during aging, which are most notable in weight-bearing lower limb muscle groups [36, 44]. Caloric restriction is generally required to achieve significant weight loss; typically 1/3rd to 1/4th of the lost weight is fat-free mass, a significant concern for older adults. In contrast, exercise can preserve muscle mass [45, 46] and improve muscle quality [47, 48] in older adults, but does not typically produce significant weight loss by itself [49]. As such, identifying interventions designed to maximize functional improvement while limiting muscle mass loss during weight loss is a current challenge. 

**Figure 3:**
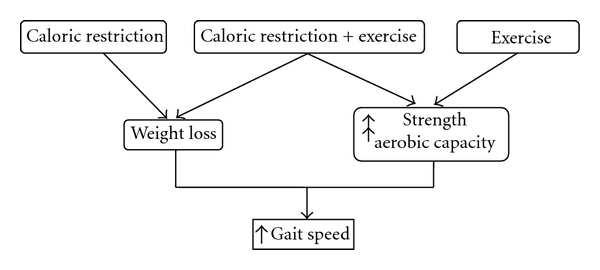
Model illustrating the potential effects that single- and multicomponent interventions have on gait speed.

Weight loss through diet and exercise interventions may improve mobility through several mechanisms (see Figure 3). First, weight loss through caloric restriction could lessen the mechanical load on weak joints and muscles, thereby improving mobility. For example, Messier et al. found a direct association between weight loss and attenuation of knee joint moments and forces during walking in overweight and obese older adults with knee osteoarthritis following an 18-month, weight-loss intervention [21]. Specifically, this study found that each pound of weight lost was associated with a four times reduction in the load exerted on the knee per step, which would equate to more than 4,800 pounds less in compressive load per mile walked. Messier et al. [50] also looked at a subset of these participants dividing them into high, low, and no weight loss groups (groups lost 10.2%, 2.7%, and 0%, resp.). This study provided evidence that large weight loss in overweight and obese older adults reduces maximum knee compressive forces significantly more than small weight losses. Collectively, these results suggest that weight loss achieved through lifestyle changes can induce biomechanical improvements in knee joints loads during walking, thereby reducing or limiting mobility impairments. Below, we review the effects of lifestyle interventions involving both dietary and exercise modification on changes in mobility function in obese, older adults. 

### 4.1. Calorie Restriction

Negative energy balance can be achieved by reducing energy intake or increasing energy expenditure. Calorie restriction (CR) has consistently been shown to extend lifespan and reduce age-related diseases in numerous species [51]. Emerging findings also suggest that CR can produce health benefits for nonobese humans (BMI range = 23.5–29.9), such as reductions in body weight and whole body fat mass [52] and beneficial effects on “biomarkers of aging” (i.e., fasting insulin level, core body temperature) in overweight individuals (BMI range = 25.0–29.9) [53]. The effects of CR on changes in mobility function in obese, older adults, however, are less well known. Two recent studies described below discuss the effect that diet-alone interventions may have on mobility and physical function in obese, older adults. 

A recent study conducted by Avila and colleagues evaluated the effect of a 10-week dietary intervention compared to a diet plus resistance training (RT) intervention on physical functioning in obese, older adults [54]. Both groups significantly increased walking speed, documented by reduced time to complete the 400-meter walk test (reduction in time for diet: −36 sec; diet plus RT: −40 sec). These results suggest that diet alone may improve global mobility function in obese, older adults; however, these effects may be heightened when a dietary and exercise intervention are combined.

In an even more recent study, Villareal and colleagues conducted a clinical trial evaluating changes in physical function in obese, older adults who were randomized into a weight management program, exercise training program, weight management plus exercise training program, or control group [55]. The diet-only weight management program consisted of a balanced diet producing an energy deficit of 500 to 750 kcal per day from participants' daily energy requirements. The primary measure of physical performance was the modified Physical Performance Test which included several standardized tasks such as walking 50 feet, standing up from a chair, lifting a book, and climbing one flight of stairs. Compared to the control group, the diet-only weight management group significantly improved their score on the Physical Performance Test, increased peak oxygen consumption, lost more fat mass, and reduced the time needed to complete an obstacle course. It is important to note, however, that, while the weight management program improved physical function in obese, older adults, the combination of diet and exercise resulted in the greatest improvements in physical function. These results are discussed further below.

### 4.2. Exercise Training

Epidemiological data have clearly demonstrated a dose-response pattern for physical activity to reduce the risk of mobility limitations [56, 57]. Additionally, many small clinical trials have reported beneficial effects of aerobic exercise on physical capacity and gait speed [58, 59]. For example, Brown and Holloszy conducted a series of studies that clearly demonstrate that aerobic exercise alone is effective at increasing walking velocity through improvements from cardiovascular capacity in healthy lean, older adults [60]. Despite this knowledge, there has been little carryover to specific studies on obese, older adults. The studies discussed below provide information regarding general mobility adaptations to resistance and/or aerobic interventions in obese and nonobese older adults. 

Resistance exercise is the best method of improving skeletal muscle performance. Accumulating evidence suggests that resistance training increases muscle strength in older adults, thereby greatly attenuating the losses of strength, power, and muscle mass that occur during aging [61, 62]. Moreover, resistance training has robust effects on the muscle strength and overall physical function of older adults [63, 64]. Further, recent studies have found that resistance-training interventions produce clinically meaningful improvements in gait speed in older adults with mobility limitations and that increased muscular power contributed to these improvements [65]. 

Numerous studies provide evidence that skeletal muscles, even among frail older adults, adapt vigorously to resistance training with marked myofibre hypertrophy [62]. This is an important finding considering that reductions in muscle power and strength are, at least in part, related to preferential type II myofibre atrophy [66]. For example, a study by Charette and colleagues found that a 12-week resistance training program in older women (mean age 69 years ± 1 year) significantly increased the cross-sectional area of the type II muscle fibers in comparison with an educational control group [61]. In another study, Fiatarone and colleagues examined the effects of an 8-week, high-intensity resistance program in ten frail, institutionalized older adults (90 years ± 1 year). The researchers found that mid-thigh muscle area increased by 9.0%  ±  4.5% and that mean tandem gait speed improved by 48% [62]. In addition, participants had an average strength gain of 174%. In another recent study, Ferri and colleagues assessed changes in muscle strength and power following a 16-week resistance program in a population of men between the ages of 65 and 81. The researchers found not only an increase in maximal force production but also a significant increase in muscle power [63]. These studies provide compelling evidence that a progressive resistance training program can reduce muscle atrophy, improve gait speed, and increase muscle power.

Combination exercise training programs are often used to meet the current physical activity recommendations by the ACSM/AHA which state that older adults should take a multifactorial approach to enhancing physical activity by performing aerobic, strength, and flexibility exercise. Findings from recent trials support the efficacy of interventions that incorporate both aerobic and resistance training (with or without dietary intervention) for improving physical function in obese, older adults [55]. Another recent clinical trial sought to determine whether combining aerobic and resistance exercise compared to either modality alone (i.e., combination versus aerobic exercise alone versus resistance exercise alone) would exert greater effects on risk factors for disease and disability [67]. Abdominally obese older men and women completed a six-month intervention consisting of either resistance exercise three times per week, aerobic exercise (treadmill walking) five times per week, resistance and aerobic exercise three times per week, or a nonexercise control. All intervention groups showed improvements in functional limitations; however, this improvement was greater for the combined exercise group compared to the aerobic-only exercise group. Furthermore, the combined and aerobic-only groups lost significantly more total fat and abdominal fat than the control and resistance-only group, while skeletal muscle mass was most improved in the combined and resistance exercise group. 

However, there is contradictory information presented by Manini and colleagues that demonstrated that one year of aerobic and resistance exercise performed two times per week was ineffective at improving long-distance walking speed in obese men and women when compared to nonobese [68]. Despite little change in long-distance walking speed (400 meters), obese individuals did manage to gain clinically significant improvements in a short performance battery of physical function that includes chair rises, balance tests, and 4-meter walking test. 

The most recent data by Villareal and colleagues help to confirm the independent effects of exercise (aerobic + resistance exercise) on mobility and physical function in obese men and women [55]. In this study, exercise alone (3 times per week) resulted in significant improvements in a battery of functional tasks that included walking 50 feet, climbing one flight of stairs, and performance on a Romberg balance test—a motor coordination test that requires subjects to maintain static balance under progressively more difficult altered base of support conditions. Additional functional tasks that improved in the Villareal study included putting on and removing a coat, picking up a penny, standing up from a chair, and lifting a book. Collectively, these effects were greater than a concurrent control group and exceeded the improvements in a group who completed a diet-only intervention. 

Collectively, these studies suggest that exercise alone can have robust effects on physical function in obese, older adults. However, these effects might not exceed those found with nonobese individuals. Therefore, an exercise program combining aerobic and resistance exercise provides the most beneficial health effects in obese older adults.

### 4.3. Multicomponent Interventions

Many lifestyle interventions designed to improve physical function in obese, older adults have typically included both a dietary component and a physical activity program, and thus can be classified as a *multicomponent intervention*. Because studies that intervene with caloric restriction plus exercise generally demonstrate beneficial effects on long-distance walking ability [4], a multicomponent intervention that includes caloric restriction may be needed to optimize the benefits of moderate intensity physical activity in obese, older adults. A relatively small number of studies have examined the effects of multicomponent interventions that combine dietary restriction plus exercise in obese, older adults. 

Findings from recent trials support the effectiveness of multicomponent (i.e., diet plus aerobic plus resistance exercise) interventions for improving mobility function in obese, older adults. For example, the addition of resistance training to a diet plus aerobic exercise program can attenuate the loss of skeletal muscle during weight loss in adults aged 65 and older [69]. Diet plus supervised resistance and aerobic exercise regimens have also been found to substantially improve mobility (i.e., walking speed) and function in obese, older adults [70, 71]. A recent study examined the effects of a 24-week multicomponent intervention compared to a Successful Aging Educational Control group on changes in mobility function in overweight, older women with moderate functional impairments. Participants in the weight loss plus exercise (WL + E) intervention condition were instructed to reduce their caloric intake by 500 to 1000 kcal/day and to attend weekly group-based counseling sessions, as well as three center-based supervised exercise sessions per week. The exercise regimen included an aerobic phase (15 minutes), a resistance-training phase (15 minutes), a second aerobic phase (15 minutes), and a cool-down phase (15 minutes). This intervention resulted in substantial improvements in mobility (i.e., increase in walking speed = 0.15 meters/second) in this high-risk population [72]. Participants in the Successful Aging Educational Control condition received weekly lectures on a variety of health topics relevant to older adults. Noteworthy, walking speed among participants in this group did not change. In a recent study, Avila et al. [54] examined the effect of a 10-week moderate intensity resistance training program combined with diet-induced weight loss on body and muscle composition and physical function in obese, older adults. The combination of resistance training and diet was found to be more effective than diet alone in producing fat loss, reducing intermuscular adipose tissue, and improving strength. However, no differences were found between groups on measures of physical function. 

Another recent trial investigated the independent and combined effects of dietary and exercise interventions [55]. For this 52-week study, participants were assigned to a control group, caloric restriction group, exercise group, or a caloric restriction plus exercise group. The exercise intervention included both aerobic and resistance training components. While physical function (i.e., Physical Performance Test) improved for all the intervention groups compared to the control group, the diet plus exercise group exhibited significantly better physical function compared to the diet-only and exercise-only groups. Additionally, the diet plus exercise group lead to improved peak oxygen consumption, strength, balance, and gait speed compared to all other groups. These findings suggest that multicomponent weight loss interventions can significantly improve mobility and physical function in obese, older adults.

## 5. Conclusions

Obesity is a major health concern in most developed countries. When combined with advance age, the detrimental health effects of obesity are magnified. These negative effects manifest in poor physical performance that can be captured with a simple measurement of gait speed. The use of gait speed not only provides a marker of physical performance but also represents a strong predictor of longevity. As such, we have presented gait speed as a surrogate endpoint that estimates the effect of lifestyle interventions on longevity. Importantly, there is now extensive evidence demonstrating that lifestyle interventions involving modification of dietary and exercise patterns are effective in producing clinically significant improvements in gait speed. While interventions emphasizing either modification of diet or exercise have beneficial effects on gait speed, the benefits are optimized with multicomponent interventions. Therefore, interventions involving both diet and exercise may hold the greatest potential for improving mobility and potentially increasing longevity in obese, older adults.
